# VEGF promotes cartilage angiogenesis by phospho-ERK1/2 activation of Dll4 signaling in temporomandibular joint osteoarthritis caused by chronic sleep disturbance in Wistar rats

**DOI:** 10.18632/oncotarget.14874

**Published:** 2017-01-28

**Authors:** Yabing Dong, Gaoyi Wu, Ting Zhu, Hongyu Chen, Yong Zhu, Guoxiong Zhu, Fabin Han, Huaqiang Zhao

**Affiliations:** ^1^ School of Stomatology, Shandong University, Wen Hua Xi Lu, Jinan City 250012, Shandong Province, China; ^2^ Shandong Provincial Key Laboratory of Oral Tissue Regeneration, Wen Hua Xi Lu, Jinan City 250012, Shandong Province, China; ^3^ Department of Stomatology, Jinan Military General Hospital, Shi Fan Lu, Jinan City 250031, Shandong Province, China; ^4^ Center for Stem Cells and Regenerative Medicine, The Affiliated Liaocheng Hospital, Taishan Medical University, 252000, Shandong Province, China

**Keywords:** chronic sleep disturbance, angiogenesis, TMJ-OA, VEGF

## Abstract

Chronic sleep disturbance (CSD) has been linked to the development of temporomandibular joint osteoarthritis (TMJ-OA). While the pathogenesis of TMJ-OA is unclear, recent studies indicate that osteochondral angiogenesis is important. We developed a rat model of CSD induced TMJ-OA to investigate the changes caused by sleep disturbance and to correlate them with vascular invasion in the TMJ. We found pathological alterations and an increased microvessel density in the rat TMJ following CSD. VEGF, Dll4 and p-ERK1/2, the expression of angiogenic factors, were highly expressed in the rat mandibular condylar cartilage and their expression increased with CSD. Furthermore, we show that VEGF-induce activation of ERK1/2, which in turn, increases Dll4 expression. Together, our results suggest that CSD can cause OA-like pathological alterations in the rat TMJ by increasing angiogenesis.

## INTRODUCTION

The temporomandibular joint (TMJ) is one of the most common sites invaded by osteoarthritis (OA), a degenerative joint disease. Temporomandibular joint osteoarthritis (TMJ-OA) is characterized by cartilage degradation, subchondral bone remodeling, chronic pain and joint dysfunction [1, 2]. OA can increase the risk of psychological stress as ongoing pain and sleep problems sometimes trigger functional disability and depression [3]. The increased daily stress experienced by OA sufferers can lead to chronic sleep disturbance (CSD) related disorders. Moreover, CSD can cause hormonal and neurotransmitter changes within the body, which could be closely related to the pathogenesis of temporomandibular disorders (TMDs). Indeed, an increasing number of academics view CSD as important for the occurrence and development of TMJ-OA.

While the pathogenesis of TMJ-OA remains unclear, recent studies indicate osteochondral angiogenesis may contribute to the development of OA [4–7]. While healthy articular cartilage typically produces angiogenic inhibitors, pro-angiogenic factors, such as vascular endothelial growth factor (VEGF), may be involved in TMJ-OA pathogenesis. Indeed, VEGF was shown to be upregulated in the chondrocytes of mandibular condyles with OA changes [8], and can lead to the formation of new vessels in OA cartilage [9]. VEGF binds to its receptor (VEGFR-2), leading to ERK1/2 activation, which promotes endothelial cell survival, proliferation, migration and invasion [10]. Moreover, ERK helps induce matrix metalloproteinase, which aid degradation of the extracellular matrix during initiation and progression of TMJ-OA [11]. In addition to VEGF, the Notch receptors and ligands (Jagged and Delta-like) stimulate vascular formation [12]. For example, the delta like ligand 4 (Dll4) is an important component of the Notch pathway that contributes to stem cell self-renewal and vascular development. Indeed, the activation of Dll4–Notch by VEGFR-2 and the repression of VEGFR-2 expression downstream of Notch activation are seen as two crucial processes regulating endothelial sprouting and angiogenesis [13, 14], while there are few papers investigating the function of VEGF and Dll4 in the process of TMJ-OA.

Our preliminary studies confirmed that CSD can cause changes in the microstructure of TMJ cartilage, by altering pain-related factors, inflammatory cytokines, the NF-kb pathway and the ERK pathway [15–17]. However, little information is available about CSD and cartilage angiogenesis in the condylar cartilage in TMJ-OA. Here, we examined the relationship between CSD and cartilage angiogenesis in TMJ-OA. In particular, we developed a rat CSD model to investigate the role of the VEGF pathway in the development of TMDs, including TMJ-OA. We observed the expression and functional alterations of VEGF, Dll4 and ERK1/2 in the rat mandibular condylar cartilage (MCC) during CSD. We also investigated the relationship between Dll4 expression and ERK1/2 activation that are induced by VEGF, using the MEK/ERK inhibitor U0126.

## RESULTS

### CSD increases serum corticosterone and adrenocorticotropic hormone concentrations in the rat

**Figure 1:**
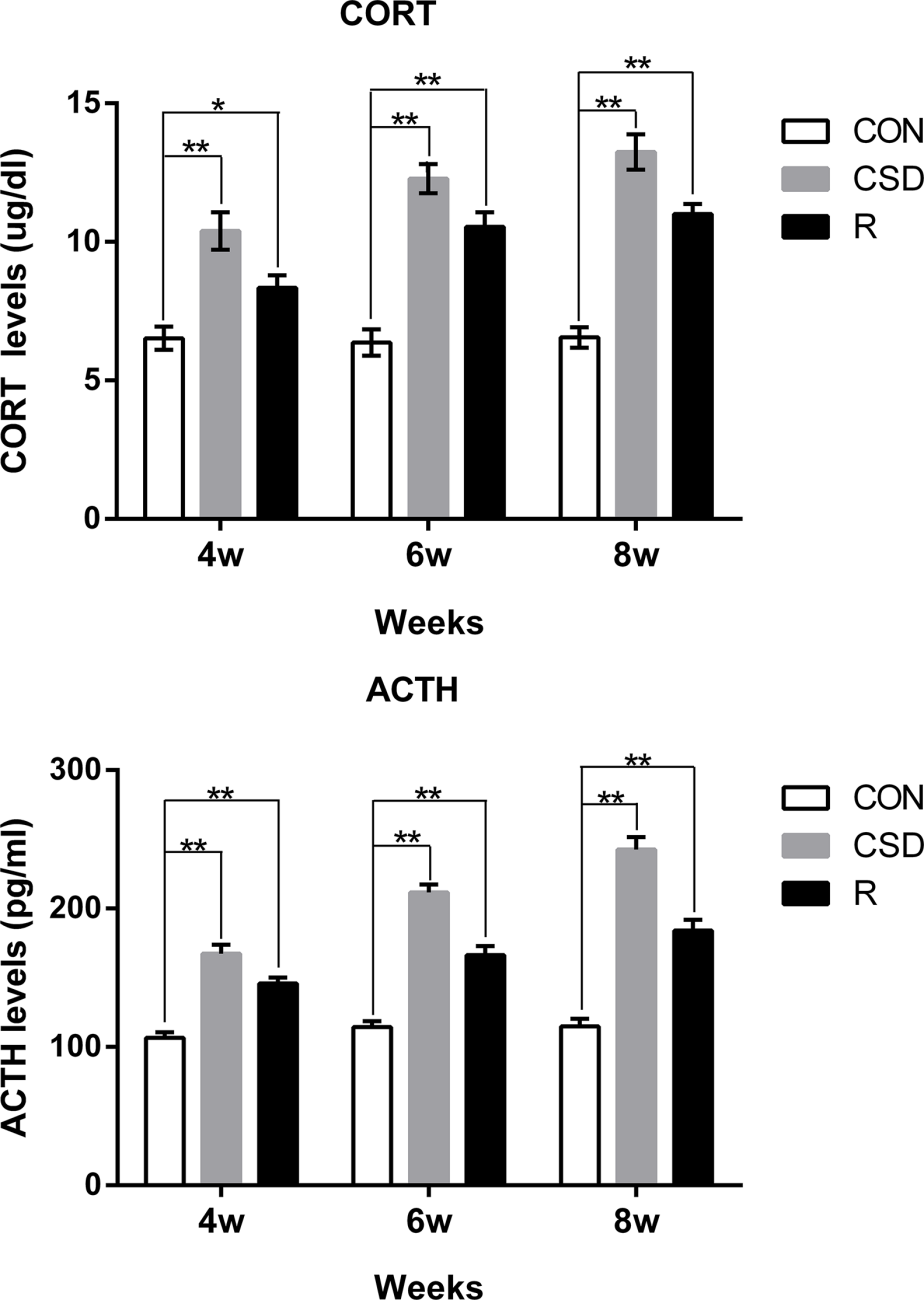
Serum levels of corticosterone (CORT) and adrenocorticotropic hormone (ACTH) from rats Bars represent the mean ± SD of each group. Significant differences between the groups are marked with asterisks (*P < 0.05, **P < 0.01). (R: group).

We successfully established a CSD rat model using the modified multiple platform method (MMPM), a well-established method for introducing sleep disturbance in rats. Rats were randomly divided into three groups (*n* = 60 per group): the control (CON) group, the chronic sleep disturbance (CSD) group using the MMPM, and the recovery group. Rats in the recovery group had undergone CSD for a period of time (i.e., 4, 6, or 8 weeks) and then the sleep disturbance was removed. To verify that the rats subjected to MMPM were under CSD stress, we analyzed the serum corticosterone (CORT) and adrenocorticotropic hormone (ACTH) levels. Both CORT and ACTH levels in the CSD group were significantly higher than the CON group (*P <* 0.05) after 4, 6, and 8 weeks (Figure 1). CORT and ACTH levels in the recovery group were also significantly higher than that of CON group (*P <* 0.05), even though the rats in the recovery group had returned to normal sleeping patterns. This suggests that the rats need a much longer recovery time for their CORT and ACTH to return to normal.

### CSD causes OA pathological alterations in the Rat TMJs

**Figure 2:**
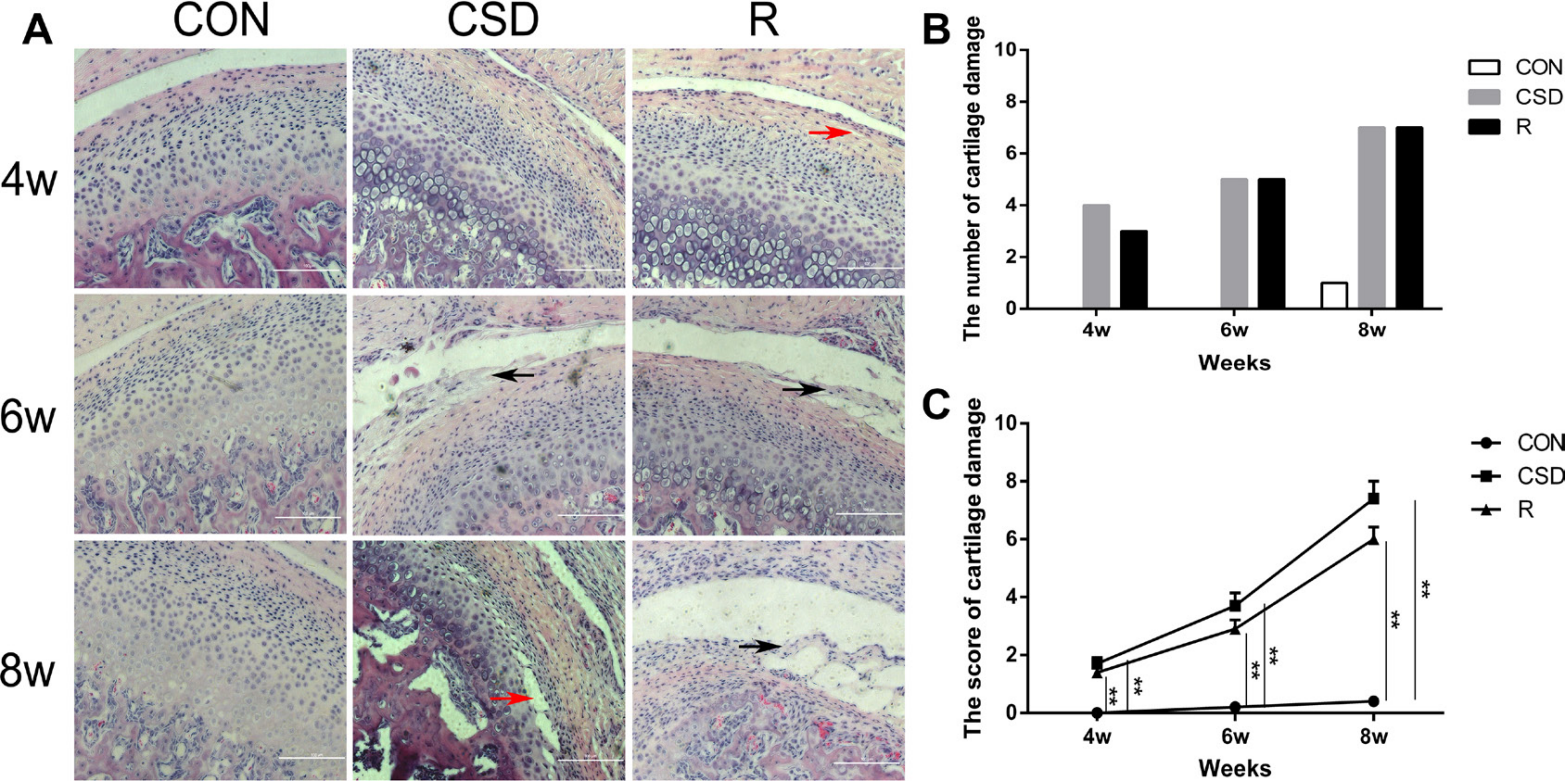
Rat TMJ sections stained with hematoxylin and eosin (**A**) Central condyles of the CON, CSD and recovery group at 4, 6, 8 weeks after sleep disturbance (original magnification: ×200, scale bar = 100 μm). (**B**) The number of cartilage damage for rats in the CON, CSD and recovery group (*n* = 10 per group) at 4, 6, 8 weeks after sleep disturbance. (**C**) The score of cartilage damage in the CON, CSD and recovery group (*n* = 10 per group) at 4, 6, 8 weeks after sleep disturbance. Bars represent the mean ± SD of each group. Significant differences between the groups are marked with asterisks (*P < 0.05, **P < 0.01). (R: group).

We examined histologically the intermediate zone of the articular disk and the corresponding condylar cartilage using haematoxylin and eosin (HE) staining. In the CON group, the TMJ appeared normal, with a smooth condylar cartilage surface, and chondrocytes homogenously distributed throughout the cartilage. There were no obvious pathological changes in the CON group. On the other hand, the histological features of the TMJ in the CSD group, which were subjected to sleep disturbance, were consistent with the known histology of TMJ-OA (Figure 2A). Moreover, in the CSD group, the coronal sections demonstrated some histological features of OA, such as articular surface irregularities and cleft formation (Figure 2A; red arrow). In addition, the fibrous articular surfaces of the condylar cartilages became visibly tougher in the CSD group, and a debonding fibrous layer (Figure 2A; black arrow) appeared in the majority of samples after 6 and 8 weeks of sleep disturbance. The degree of damage at 8 weeks is the most serious in this study, involving all layers of the TMJ condylar cartilage. Although the histopathological changes (such as articular surface irregularities and, cleft formation) observed in the recovery group were similar to the CSD group, the damage level of recovery group was less than the CSD group. In addition, both the number and score of cartilage damage (according to the scored method of evaluating cartilage damage [18] increased in the CSD group over time compared to controls (Figure 2B and 2C). In order to evaluate the level of cartilage damage, we scored the cartilage damage. The score of the CON group at 8 weeks was 0.60 ± 0.70 (mean ± SD). Compared with the CON group, the score significantly increased (*P <* 0.05) in the CSD and recovery groups at 8 weeks (7.40 ± 1.90 and 6.00 ± 1.33, respectively; Figure 2C). Together, these findings confirm that CSD can cause OA pathological alterations in rat TMJ, which could be slightly alleviate by stopping the sleep disturbance.

### CSD increases VEGF expression in the TMJ

**Figure 3:**
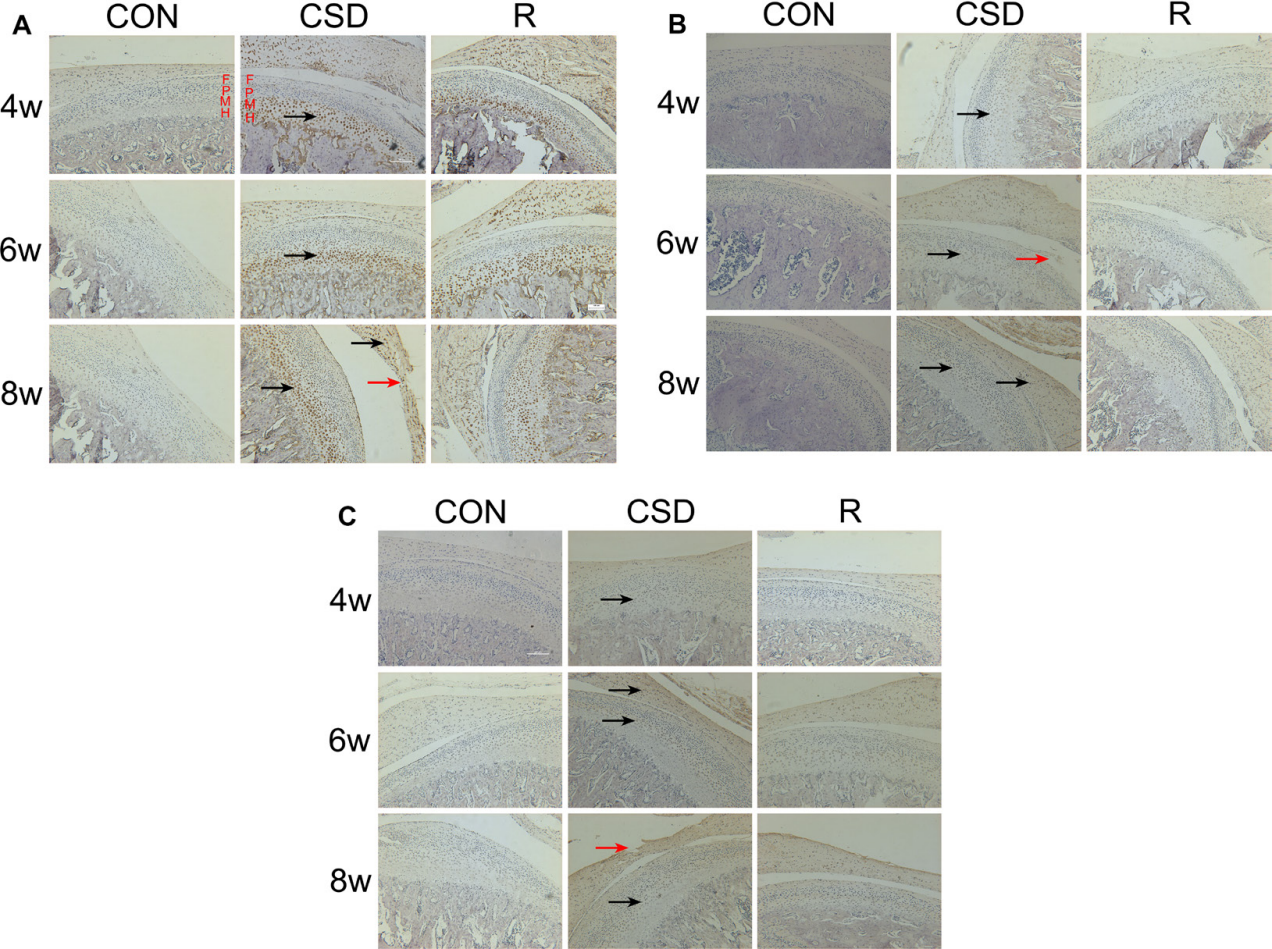
Expression of VEGF, Dll4 and p-ERK1/2 protein in the mandibular condylar cartilage of rat TMJs Representative images of immunohistochemistry staining for VEGF, Dll4 and p-ERK1/2 in the CON, CSD and recovery group (original magnification: ×200, scale bar = 100 μm). (**A**) Expression of VEGF protein in the rat TMJ mandibular condylar cartilage layers: fibrous layer (F), proliferating cell layer (P), mature cell layer (M), hypertrophic cell layer (H). VEGF-positive cells are indicated with black arrows and the damaged zone of the articular disc of TMJ is indicated with red arrows. (**B**) Expression of Dll4 protein in the rat TMJ mandibular condylar cartilage. Dll4-positive cells are indicated with black arrows and the damaged zone of the condylar cartilage is indicated with red arrows. (**C**) Expression of p-ERK1/2 protein in the rat TMJ mandibular condylar cartilage. Phosphorylated-ERK1/2-positive cells are indicated with black arrows and the damaged zone of the articular disc of TMJ is indicated with red arrows. (R: group).

We evaluated the expression of VEGF in the rat TMJ during and after CSD. Cytoplasmic staining of the VEGF protein revealed that it is mainly found in the in chondrocytes, especially in the mature (M) or hypertrophic cell layer (H) of condylar cartilage. Immunohistochemistry showed that while the CON group had no or very low numbers of VEGF-positive-chondrocytes (Figure 3A, lane 1), there was an increase in the number of VEGF positive-chondrocytes in the CSD and recovery groups (Figure 3A, lanes 2 and 3). These VEGF-positive-chondrocytes were involved in all layers of the TMJ condylar cartilage, even in the articular disc of the TMJ in the CSD group at 8 weeks. After the CSD was stopped in the recovery group, the area occupied by these VEGF-positive-chondrocytes decreased.

### CSD increases Dll4 and p-ERK1/2 expression in the TMJ

**Figure 4:**
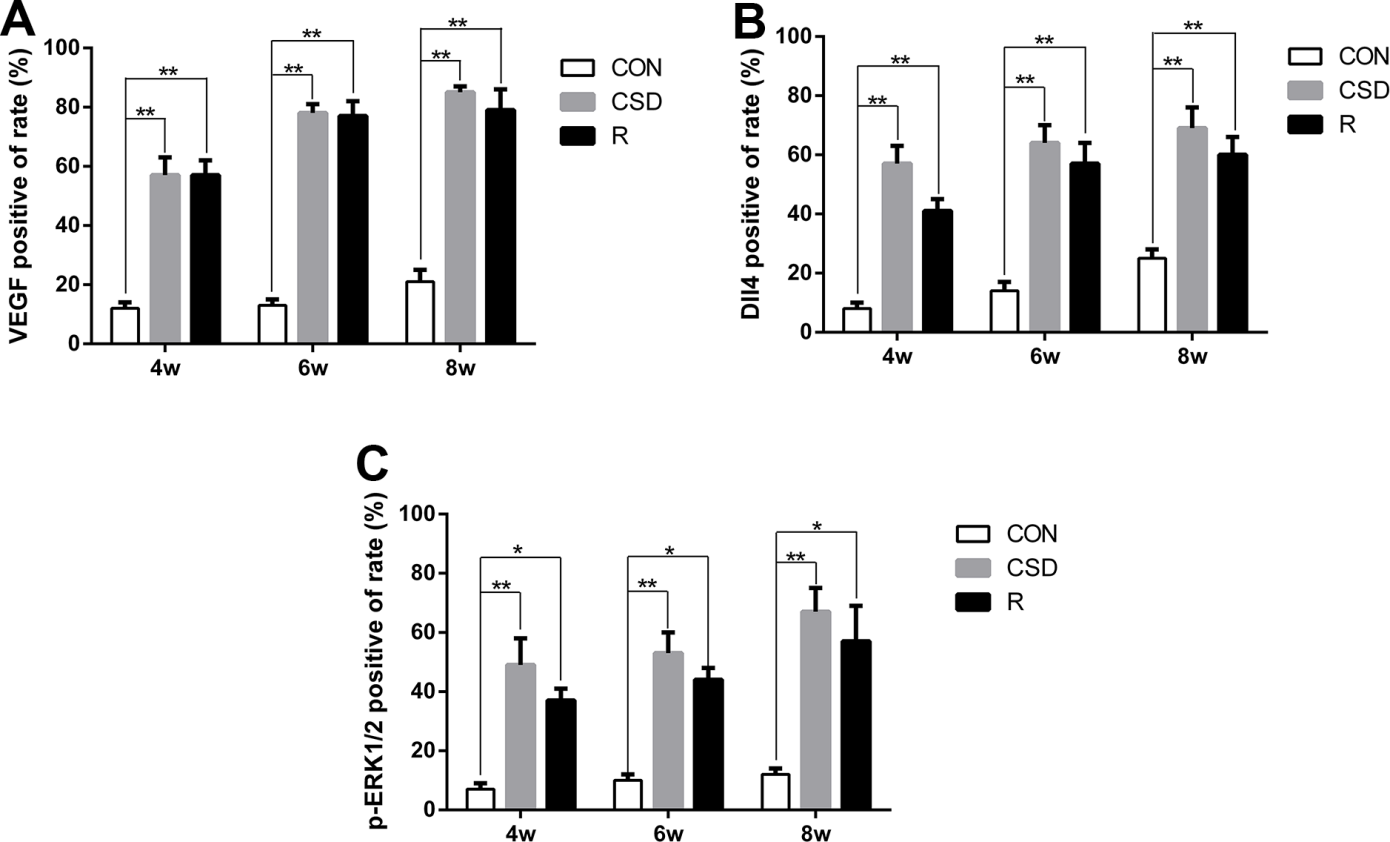
Immunohistochemical analysis of VEGF, Dll4 and p-ERK12 protein expression (**A**) Immunohistochemical analysis of VEGF protein expression. (**B**) Immunohistochemical analysis of Dll4 protein expression. (**C**) Immunohistochemical analysis of p-ERK12 protein expression. Significant differences compared with the CON group. (*P < 0.05, **P < 0.01). (R: group).

Next, we detected the expression of Dll4 (Figure 3B) and p-ERK1/2 (Figure 3C) in the TMJ in rats during and after CSD. Immunohistochemistry revealed that staining for Dll4 is predominantly within the zone of fibrous layer (F) and mature cell layer (M) (Figure 3B). On the other hand, the p-ERK1/2-positive-chondrocytes appeared in the zone of fibrous layer (F) and mature cell layer (M) (Figure 3C). These Dll4- and p-ERK1/2-positive-chondrocytes were also involved in the zone of the articular disc of the TMJ, in both the CSD and recovery groups. Compared to control cartilage tissue (Figure 3B and 3C, lane 1), the Dll4- and p-ERK1/2-positive-chondrocytes were clearly visible in CSD group and recovery group (Figure 3B and 3C, lane 2, 3). The expression of VEGF, Dll4 and p-ERK12 markedly increased over time in the CSD group compared with controls. Figure 4 showed the Immunohistochemical analysis of VEGF, Dll4 and p-ERK12 protein expression. Interestingly, we found that the level of expression of Dll4 protein was similar to that of the p-ERK1/2 protein, no matter which layer of condylar cartilage was examined.

### CSD increases VEGF, p-ERK1/2 and Dll4 expression in the mandibular condylar cartilage

**Figure 5:**
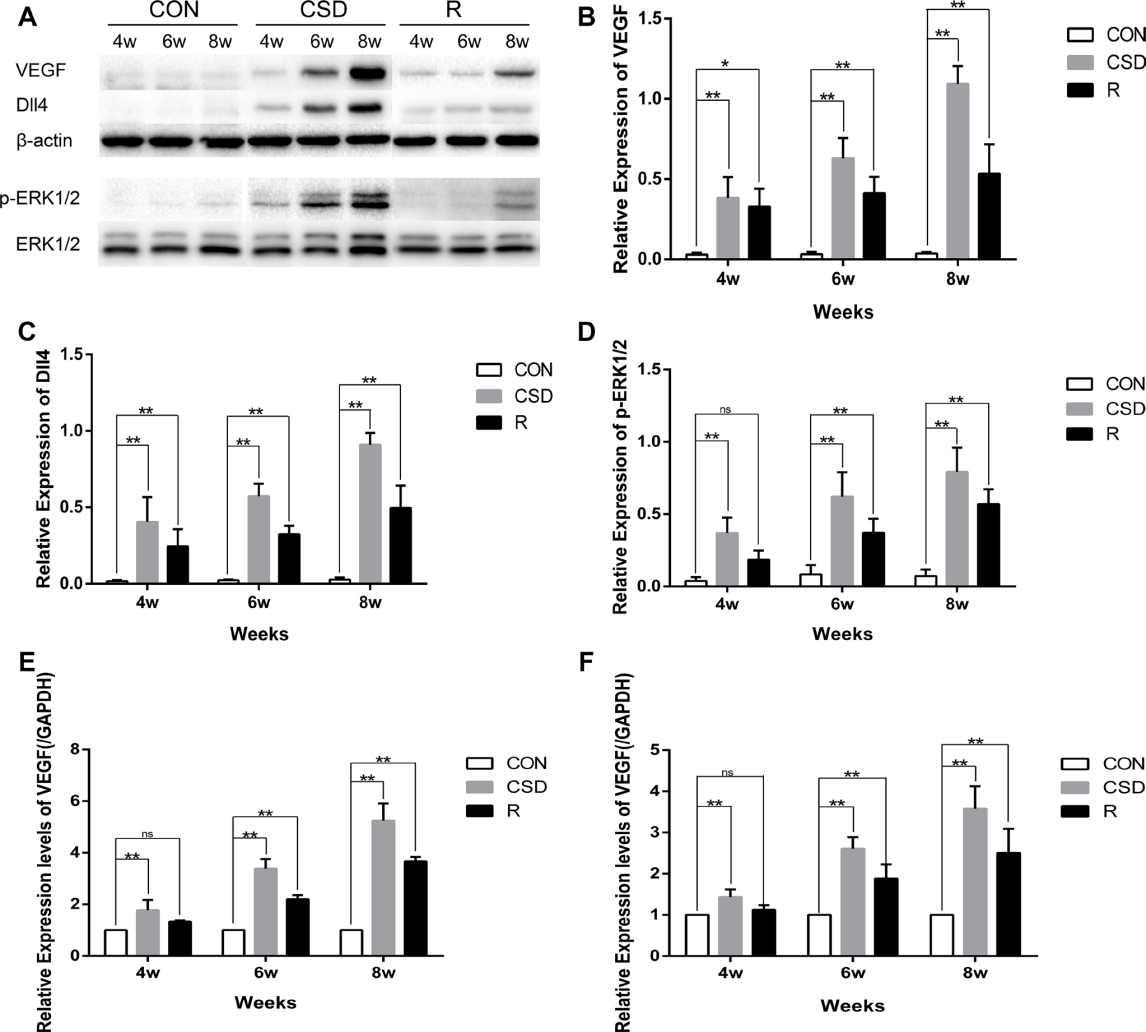
Western blot and RT-qRCR showing the change in expression of VEGF, Dll4 and p-ERK1/2 (**A**) Western blot with 3 replicates showing the change in expression of VEGF, Dll4 and p-ERK1/2. (**B**–**D**) The relative protein levels of p-ERK, ERK, MMP-1, MMP-3, and MMP-13 in different groups. (**E**) Relative VEGF mRNA levels in the condylar cartilage. (**F**) Relative Dll4 mRNA levels in the condylar cartilage. The 2^–ΔΔCt^ method was adopted with GAPDH as the reference gene. Bars represent the mean ± SD of each group (*n* = 10). Significant differences between the groups are marked with asterisks (*P < 0.05, **P < 0.01). For loading controls, the membrane was stripped and then incubated with anti-ERK (1:1000) or anti-β-actin. Significant differences between the groups are marked with asterisks (*P < 0.05, **P < 0.01); ns: no statistical significance. (R: group).

Many studies have investigated the mechanism of VEGF-induced activation of p-ERK1/2 and upregulation of Dll4 expression in tumors, but few studies have focused on this phenomenon in the TMJ. We found that VEGF was significantly increased in the CSD group compared with the control group after 4, 6, and 8 weeks of sleep disturbance (Figure 5A and 5B). Increased expression of VEGF was also found in the recovery group compared to the control group, but this level was lower than that in the CSD group. Therefore, by removing the sleep disturbance, the level of VEGF decreased.

As p-ERK1/2 and Dll4 have been suggested to play a critical role in the development of TMJ-OA, we also detected the expression of p-ERK1/2 and Dll4 in TMJs by western blot. The expression of p-ERK1/2 and Dll4 markedly increased over time in the CSD group compared with controls (Figure 5A, 5C and 5D). To explore the correlation between VEGF and Dll4, we determined the relative expression of the mRNA levels in the CSD and recovery groups compared to controls. RT-qPCR revealed that the expression levels of VEGF and Dll4 were significantly higher in the CSD group compared with the control group (Figure 5E and 5F; *P <* 0.01). The VEGF mRNA levels also significantly increased in the recovery group compared to controls (Figure 5E; *P <* 0.01), but to a lower level than the CSD group. Together, these results suggest that the VEGF-induced activation of p-ERK1/2 and upregulation of Dll4 expression in rat condylar cartilage after the rats were subjected to sleep disturbance. Therefore, VEGF, Dll4 and p-ERK1/2 may be involved in TMJ-OA pathogenesis.

### CSD can promote angiogenesis in the TMJ

**Figure 6:**
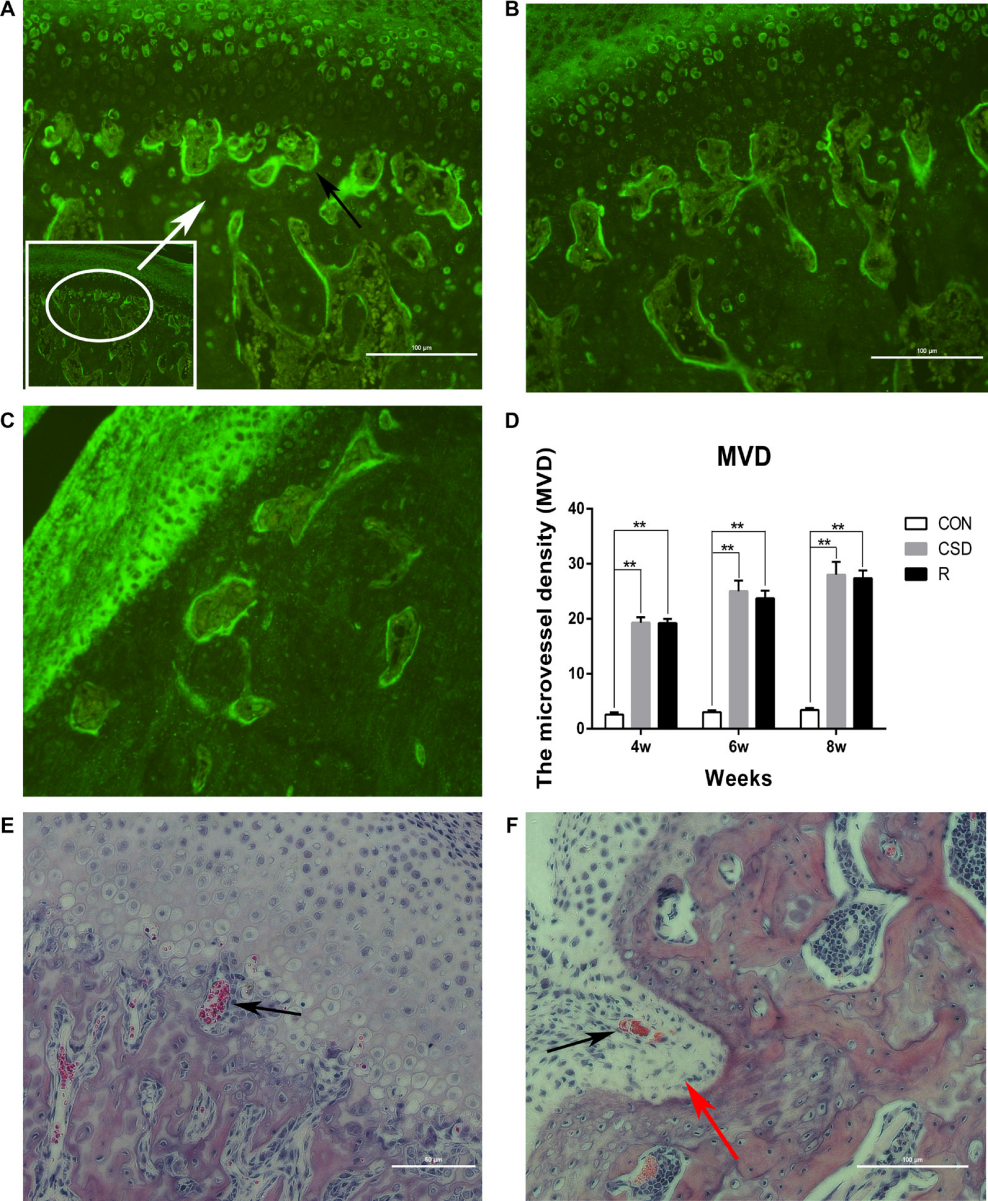
Angiogenesis in the TMJ of the CON, CSD and Recovery group Blood vessels are indicated by black arrows. (**A**) Representative immunofluorescence image of a vascular channel at the osteochondral junction in CSD group. (**B**) Representative immunofluorescence image of a vascular channel at the osteochondral junction in R group. (**C**) Representative immunofluorescence image of a vascular channel at the osteochondral junction in CON group. Immunofluorescence for the endothelial marker CD105 shows the sites of angiogenesis. The hot spot for microvessel formation is indicated by the white arrow (original magnification: ×200, scale bar = 100 μm). (**D**) The microvessel density (MVD) in the different groups (*n* = 10 per group) at 4, 6, 8 weeks after sleep disturbance. Bars represent the mean ± SD of each group. Significant differences between the groups are marked with asterisks (*P < 0.05, **P < 0.01). (**E**, **F**) Haematoxylin and eosin staining of a vascular channel (original magnification: ×200, scale bar for E = 50 μm, scale bar for F = 100 μm) the red arrow indicates that the condylar cartilage stretches into the subchondral bone. (R: group).

We found an increased number of blood vessels in the TMJ in the CSD and recovery group using immunofluorescence and HE staining (Figure 6). In the control group, vascular channels were clearly confined and did not invade the osteochondral junction. The vascular channels arising from the subchondral bone are evident at the osteochondral junction (black arrows in Figure 6). A small number of blood vessel breached into the non-calcified cartilage, located entirely within the cartilage in the CSD group with long-lasting (8 weeks) sleep disturbance (Figure 6F). We also calculated the microvessel density (MVD) in the TMJ in the different groups and found that vascular invasion at the osteochondral junction in the CSD and recovery group was significantly increased compared to controls (Figure 6D; *P <* 0.01). The density of blood vessels located in the osteochondral junction in the CSD group (MVD = 19.3 ± 0.989) was greater than in the CON group (MVD = 9.2 ± 0.68) at 4 weeks (Figure 6D; *P <* 0.001). Moreover, the MVD of the CSD group at 8 weeks (28 ± 2.3) was higher than that at 4 weeks (19.3 ± 0.99) (Figure 6D; *P <* 0.05). Finally, the MVD of the R group was not reduced upon returning to normal sleeping time. These results show that the number of blood vessels breaching into the osteochondral junction of the TMJ increased after CSD. Therefore, sleep disturbance can promote angiogenesis in the TMJ.

### VEGF induces expression of Dll4 and p-ERK1/2 in mandibular condylar cartilage cells

**Figure 7:**
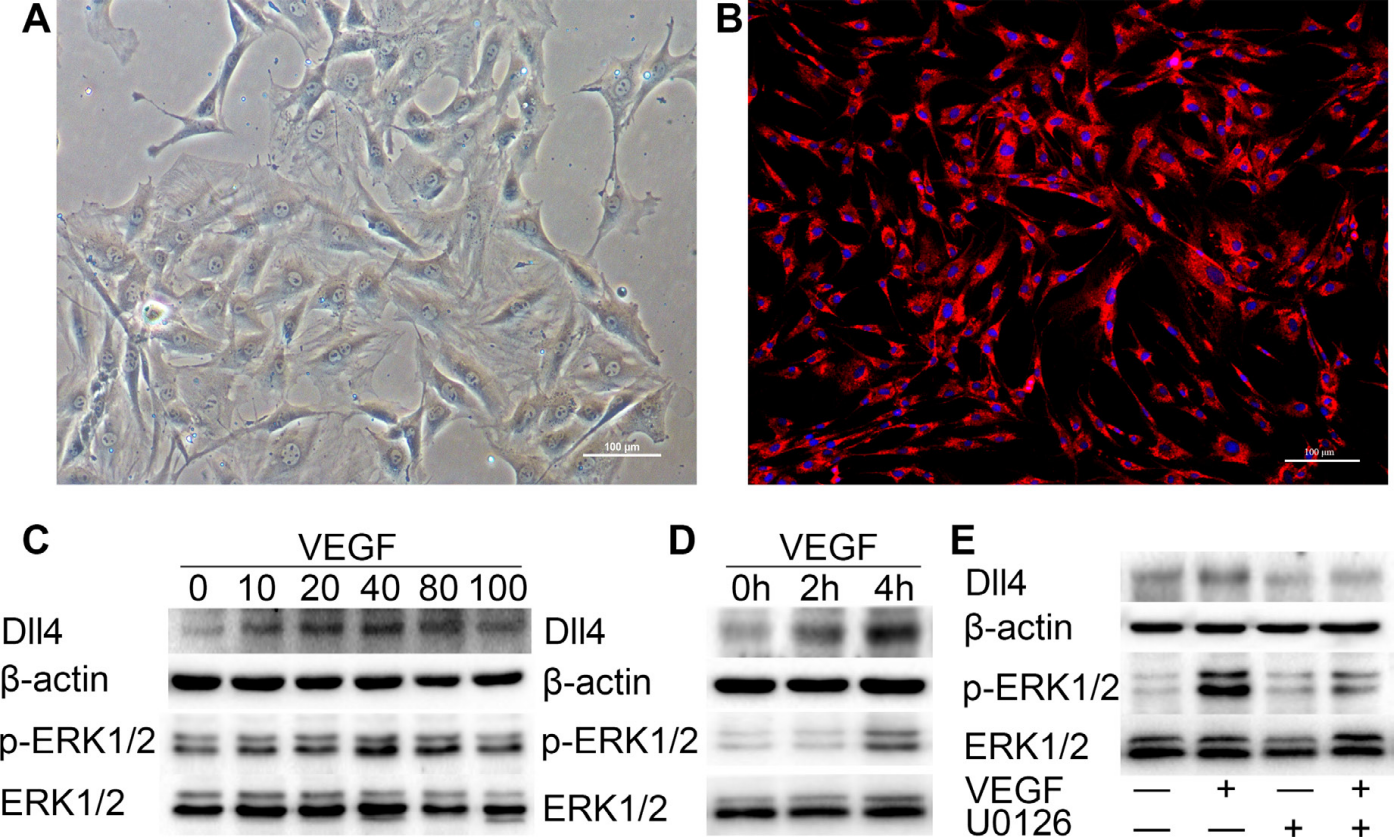
VEGF induces high expression of p-ERK1/2 and Dll4 in MCC cells (**A**) P3 of MCC cells. (Original magnification: ×100, scale bar = 100 μm). (**B**) All cells were positive for type II collagen Immunofluorescence analysis (original magnification: ×100, scale bar = 100 μm). (**C**) Western blot with 3 replicates showing the expression of p-ERK1/2 and Dll4 in MCC cells was rapidly induced by VEGF in a concentration-dependent fashion (concentration of VEGF was 0 ng/ml, 10 ng/ml, 20 ng/ml, 40 ng/ml, 80 ng/ml and 100 ng/ml, respectively). (**D**) Western blot showing the expression of p-ERK1/2 and Dll4 in MCC cells was induced by VEGF (10 ng/ml) in a time-dependent manner (h: hour). (**E**) Treatment with the MEK inhibitor U0126 significantly suppressed VEGF-induced Dll4 expression in MCC cells. MCC cells were pretreated with U0126 at 10 μM for 1 h and then treated with VEGF for 4 h before performing western blots on the MCC cell extracts. As a control, the membrane was stripped and incubated with β-actin and ERK1/2 (—: MCC cells were pretreated without U0126 or VEGF. +: MCC cells were pretreated with U0126 or VEGF). (R: group).

After we developed a primary culture of MCC cells (Figure 7A), we performed Immunofluorescence analysis with anti-collagen type II antibodies to identify the MCC cells. The cells only showed a positive reaction with the anti-type II collagen antibody (Figure 7B). By screening, these MCC cells were treated with VEGF (10 ng/ml) for 2 h (Figure 7C and 7B). Treatment of these MCC cells with VEGF potently induced the p-ERK1/2 activation and Dll4 expression in a concentration-dependent manner as determined by western blot analysis (Figure 7C). Moreover, the VEGF-induced expression of p-ERK1/2 and Dll4 in MCC was time-dependent (Figure 7D). These results suggest that a high concentration of VEGF could upregulate the ERK1/2 and Dll4 expression in MCC cells.

### VEGF induced expression of Dll4 is activated by the ERK1/2 signaling pathway

Previously, we generated the p-ERK1/2 and Dll4 expression profile in the condylar cartilage of TMJ using immunohistochemistry, and the areas of p-ERK1/2 and Dll4 expression have a striking resemblance. These findings indicate that p-ERK1/2 expression seems to be associated with Dll4. Therefore, we investigated whether Dll4 expression was dependent on the activation of p-ERK1/2 in MCC cells. We found that treatment with the MEK/ERK inhibitor U0126 at 10 μM significantly suppressed the expression of Dll4 induced by VEGF (10 ng/ml) as determined by western blot (Figure 7E). Taken together, these results indicate that the VEGF-induced expression of Dll4 is activated by the ERK1/2 signaling pathway.

## DISCUSSION

Psychological factors, such as sleep disorders, psychological stress, and depression, may cause TMJ dysfunction [19–21]. We demonstrated that sleep disturbance in rats causes significant pathological changes in the TMJ. We also provide new evidence indicating that angiogenesis in the osteochondral junction is an important factor in the pathogenesis of TMJ-OA. In our rat model of CSD induced TMJ-OA, we found increased expression of VEGF, Dll4 and p-ERK1/2 in the MCC. Moreover, we showed that VEGF-induces p-ERK1/2 signaling, which in turn, increases Dll4 expression and promotes angiogenesis in the TMJ.

We successfully established a CSD model of rats using the MMPM, in which the animals do not get the normal amount of paradoxical sleep and therefore, are under psychological stress. Paradoxical sleep disturbance activates the hypothalamic-pituitary-adrenal (HPA) axis and can cause the increase of CORT and ACTH expression [17]. And it also casuse anxiety-like behavior in rats [22, 23]. Psychological stress, such as CSD, with the increase of CORT and ACTH expression, is associated with an abnormal chewing frequency [24]. With the increase in chewing frequency, the TMJ suffers excessive loading and becomes fatigued, and the TMJ surfaces can become worn. Moreover, as the development of a chronic TMD can cause psychological distress and pain, CSD may be an important mechanism in TMJ-OA.

In condylar cartilage associated with TMJ-OA, the number of blood vessels at the osteochondral junction is markedly increased in the area directly below the hypertrophic cell layer. Thus, angiogenesis in the osteochondral junction may be an important factor in the pathogenesis of OA. However, the precise molecular pathways that activate angiogenesis in the osteoarthritic joint remain unclear. We demonstrated a time-dependent change in angiogenic activity in the subchondral bone, the condylar cartilage, and the TMJ disc in our rat TMJ-OA CSD model. We found that the blood vessels invade into the non-calcified cartilage both the CSD and recovery group. And the number of vascular channels increased at the osteochondral junction. To our knowledge, this is the first report to investigate the time-dependent changes in TMJ-OA caused by chronic sleep disturbance and to correlate them with histologically observed vascular invasion.

The development of blood vessels may contribute to the degradation of the condylar cartilage by secreting cytokines that suppress chondrocyte function, or by directly disrupting the arrangement of chondrocytes. We found that after the CSD, VEGF-expressing chondrocytes were detected in the TMJ. VEGF is the primary pro-angiogenic factor involved in angiogenesis in many tissues, including cartilage [25–27]. As vascular invasion is suggested to be an early mechanism for turning cartilage into bone, these VEGF-expressing chondrocytes may promote the destruction of cartilage. However, angiogenic activity is not only determined by VEGF; p-ERK1/2 also stimulates angiogenesis [28]. Moreover, the Dll4-Notch signaling pathway is absolutely required for normal vascular development [29], and the perichondrial layer of the MCC is rich in both Notch ligands and various downstream facilitators of Notch signaling [30]. Indeed, Notch signal pathway activation in endothelial cells has previously been shown to promote capillary-like sprout formation [31]. Therefore, we investigated the expression of the angiogenic factors (VEGF, D114, and ERK1/2) over time in our rat model. We found that the subchondral bone was an important location for angiogenesis, and that the activity of a VEGF, Dll4 and p-ERK1/2 peaked at 8 weeks in the CSD group. However, the experiment was stopped at 8 weeks as the number of deaths had increased in the CSD group. This indicates that the rats may could not live with the sleep disturbance past 8 weeks.

As few studies reported the relationship between p-ERK1/2 and Dll4, we investigated whether the activation of Dll4 is associated with p-ERK1/2. First, we showed that VEGF was significantly increased in the CSD group compared with controls after 4, 6, and 8 weeks of sleep disturbance. Second, we demonstrated that the activation of p-ERK1/2 and the upregulation of Dll4 expression induced by VEGF also stimulated the angiogenic activity in rat condylar cartilage of TMJ-OA. Third, we showed that p-ERK1/2 inhibition with U0126 reduced Dll4 expression after the MCC cells were treated with VEGF. Therefore, the p-ERK1/2 mediated increase in D114 expression may be a new finding involved in osteochondral angiogenesis.

Most TMDs are self-limiting, and have reparative effects. However, in rats that had previously experienced CSD but were now recovering with normal sleep patterns, the expression of VEGF, p-ERK1/2 and Dll4 remained high compared with controls. Therefore, the repair of the TMJ following damage due to CSD may require more time. Thus, further studies are required to determine whether the pathological alterations can be reversed after the condition of CSD is stopped.

In conclusion, we revealed the pathological changes in the TMJ of rats exposed to CSD. Our findings provide important new evidence indicating that angiogenesis is an important progressive phase in the development of TMJ-OA. Therefore, minimizing sleep disturbance and inhibiting angiogenesis could potentially be used to treat TMJ-OA.

## MATERIALS AND METHODS

### Ethics approval

All experimental procedures were carried out in accordance with the regulations and institutional guidelines of the Ethics Committee at the Stomatological Hospital of Shandong University (Permit Number: GD201509). All experimental protocols were approved by the Ethics Committee at the Stomatological Hospital of Shandong University. All surgery was performed under anesthesia, and all efforts were made to minimize the animal suffering. All the animals were sacrificed by an overdose of pentobarbital sodium after the experiment.

### Experimental design

One hundred and eighty male Wistar rats (8 weeks old, weight 200 ± 20 g) were bought from the Animal Centre of Shandong University (Jinan, China), and the experiments were done in the animal experimental center at Jinan Military General Hospital. The rats were acclimatized under laboratory conditions for 1 week before the start of the experiment. Then, all rats were adapted to the CSD for an hour per day for five consecutive days. The rats were randomly divided into three groups (*n* = 60 per group): the control (CON) group, the chronic sleep disturbance (CSD) group, and the recovery (R) group. Each group was equally divided into three subgroups (*n* = 20 per subgroup), according to the observation time points (sleep disturbance for 4 weeks, 6 weeks, or 8 weeks). The CSD rats were placed on small platforms during the procedure, whereas, the CON rats were placed on a large platform. The recovery (R) group began after the rats had completed 4, 6, or 8 weeks of CSD, and the rats were placed in the cages without sleep disturbance for 1 week, and then were killed to collect the TMJs.

### Animal model for CSD

The animals were housed in 80 cm × 45 cm × 40 cm cages. The temperature was controlled at 25°C with a 12:12 h light–dark cycle (lights on at 07:00 h and off at 19:00 h). The modified multiple platform method (MMPM) was selected to induce CSD in this study. The rats were placed on the grid and could lie down without falling into the water. All rats could move around freely inside the tank by jumping from one platform to another. The rats were awakened when they touched the water as a result of muscle atonia. After the adaptation period, the rats were placed in the MMPM and subjected to sleep disturbance for 17 h (starting at 16:30 h) every day for 4, 6, 8 weeks. The animals were allowed to sleep in their individual home cages for 7 h (beginning at 09:30 h).

### TMJ collection

The CSD was performed and groups of animals were sacrificed 4, 6 or 8 weeks later, according to their subgroups. In each subgroup, the TMJs were dissected. The connective tissues were removed to expose the areas surrounding the mandibular condyle, and 10 right joints were randomly selected from each subgroup for HE staining, and 10 left joints were randomly selected for immunocytochemistry. The remaining 10 left joints were used for HE staining. The TMJs of the remaining 10 rats were prepared for the detection of protein and RNA by western blotting and RT-qPCR.

### Rat mandibular condylar cartilage (MCC) cell preparation

First, cartilage tissues from the condylar were harvested from 10 4-week-old Wistar rats. The tissues were washed thrice with phosphate-buffered saline (PBS), minced meticulously, and digested with 0.25% trypsin (Invitrogen, USA) for 10 min, and subsequently digested with 0.1% collagenase II (Invitrogen, USA) in growth medium (Hyclone, Logan, UT, USA) supplemented with 20% fetal bovine serum (FBS) (Sciencell, USA), 100 mg/mL penicillin, and 100 mg/mL streptomycin (Invitrogen, USA). Following incubation at 37°C in a humidified atmosphere of 5% CO_2_, the MCC cells were collected at intervals of 2 h by centrifugation. Then the MCC cells were resuspended with the medium, in 6 cm culture dishes. For the duration of the MCC cell culture, the medium was changed every 3 days, and in the following experiments, the P3 of the MCC cells was used.

### Serum assay

After 4, 6, and 8 weeks of sleep disturbance, blood samples were obtained from the cardiac ventricles of the CSD and CON group rats between 09:00–12:00 h under anesthesia by intraperitoneal injections of pentobarbital sodium (50 mg/kg body weight). The serum was separated by centrifugation (3000g for 10 min at 4°C) and stored immediately at −80°C for the hormone tests. The serum concentrations of corticosterone (CORT) and adrenocorticotropic hormone (ACTH) were measured by radioimmunoassay using an Access Immunoassay System (Beckman Coulter, USA) according to the manufacturer's protocols.

### Haematoxylin and eosin (HE) staining and immunohistochemistry

The isolated right TMJs were fixed in 10% buffered paraformaldehyde for 24 h, incubated with 10% EDTA at 4°C for 4 weeks and then embedded in paraffin. Sections were cut into 5 mm sagittal sections and stained with HE for histological studies.

### The scores of cartilage damage

Table 1 shows the scoring method used to score the cartilage damage. The amount of cartilage damage was estimated as the proportion of the section of the condylar cartilage of the TMJs involved (i.e., 1/3, 2/3 or > 2/3) and the cartilage score was multiplied by 1, 2 or 3, respectively, to give a final score.

**Table 1:** The scores asigned to various levels of cartilage damage

Cartilage damage	Score
Cartilage of normal appearance	0
Minimal fibrillation, superficial zone only	1
Mild, extends to the upper middle zone	2
Moderate, well into the middle zone	3
Marked, into the deep zone	4
Severe, full thickness degeneration	5

### Immunohistochemical staining

Tissue sections were prepared as described above. Endogenous peroxidase activity was inhibited by 3% hydrogen peroxide. Antigen retrieval was performed by autoclaving at 120°C for 15 min in 0.01 M citrate buffer (pH 6.0). The sections were reacted overnight with the following rabbit polyclonal antibodies (purchased from Bioworld, China): anti-VEGF (diluted 1:50), anti-Dll4 (diluted 1:50) and anti-p-erk1/2 (diluted 1:50) at 4°C. The secondary antibody, biotinylated anti-rabbit IgG, was applied for 30 min at room temperature. The sections were visualized by 3, 30-diaminobenzidine-tetrahy-drochloride (DAB). Digital images were further analyzed via Image-Pro Plus (Media Cybernetics, USA) software. Sections were then lightly counterstained with haematoxylin, dehydrated, cleared, and mounted. For each slide, three fields were counted (magnification ×200) to access the frequency of cell-positive nuclei. The protein expression was determined by the mean of calculating the percentage of immunoreactive cells among the population of cells.

### Immunofluorescence and microvessel density (MVD) count

MVD count was determined using the modified Chalkley method [32]. In brief, tissue sections were immunofluorescencently stained with an anti-CD105 antibody (Boster, China). MVD count was determined by identifying the areas of highest vascular density for each TMJ section at 100× magnification. The areas of highest vascular density (‘hot spots’) were identified at low magnification (×100). On a higher magnification (×200), a 3-point eyepiece was applied to each hot spot and orientated to permit the maximum number of the microvessel. The number of the microvessel was counted by two independent investigators, and their average number was used for analysis.

### Western blotting

Two pieces of condylar cartilage from each rat were regarded as one sample to ensure that enough protein was available for the analysis. Briefly, the condylar cartilage tissue was homogenized in ice-cold radioimmunoprecipitation assay (RIPA) lysis buffer (Beyotime, China; 1 mmol/L PMSF included) and centrifuged at 15000 rpm for 10 min at 4°C. Equal amounts of total protein for each group (15 μg protein per lane) were separated using 10% sodium dodecyl sulphate-polyacrylamide gel electrophoresis (SDS-PAGE) and then electro-transferred onto a polyvinylidene fluoride (PVF) membrane (Beyotime, China) using the Bio-Rad protein assay system (Bio-Rad, USA). Each membrane was blocked with 5% BSA in TBST buffer (Tris buffered saline and 0.1% Tween20) at room temperature for 1 h and then incubated overnight at 4°C with the following primary antibodies: anti-VEGF (diluted 1:1000,Abcam), anti-dll4 (diluted 1:1000, Santa Cruz Biotechnology), anti-ERK1/2 (diluted 1:1000, Cell signaling technology,), anti-p-ERK1/2 (diluted 1:1000, Cell signaling technology), and anti-β-actin (diluted 1:5000, Bioworld, China). The following day, the membranes were incubated with the appropriate secondary antibodies (diluted 1:1000, Beyotime, China) for 1 h at room temperature. Proteins were detected by an ECL kit (Beyotime, China). All bands from western blot analysis were analyzed using Image Lab software (version 1.6 NIH) to verify the relative level compared to the internal control, β-actin.

### Reverse transcription and real time quantitative polymerase chain reaction (RT-qPCR) analysis

Total RNA was extracted using Trizol reagent (Sangon Biotech, China) according to the manufacturer's instructions. The TMJ tissue was ground into powder in liquid nitrogen, and reverse transcription and RT-qPCR were carried out using the PrimeScriptTM RT Reagent Kit and SYBR^®^ Premix Ex TaqTM II Kit (TaKaRa, Japan) according to the manufacturer's instruction. RT-qPCR was carried out in the 7500 Real Time PCRsystem (Applied Biosystems, USA) with the following settings: 10 min of pre-incubation at 95°C followed by 40 cycles of 20 s at 95°C and 60 s at 55°C. A 25 ml reaction volume was chosen. The primers used are shown in Table 2. Melting curve analysis was carried out using the default program. After each reaction, the cycle threshold (Ct) was recorded when the amplification curve reflected the exponential kinetic measurements. The 2^–ΔΔCt^ method was adopted with GAPDH as the reference gene.

**Table 2:** Primers used for RT-qPCR

Primer name	Sequence
VEGFa (forward)	5′-CCGGTTTAAATCCTGGAGCG-3′
VEGFa (reverse)	5′-TTTAACTCAAGCTGCCTCGC-3′
Dll4 (forward)	5′- GCCCAGACTCCATCCTTACA −3′
Dll4 (reverse)	5′-CCTGCTAAATGCCAGACTCC-3′
GAPDH (forward)	5′- ATGATTCTACCCACGGCAAG-3′
GAPDH (reverse)	5′-CTGGAAGATGGTGATGGGTT-3′

### Statistical analysis

All data were expressed as means ± standard Deviation (SD) of the mean. Statistical evaluations of experimental data were performed with SPSS for Windows (IBM SPSS Statistics Version 20.0 Chicago, IL, USA), and Two-way ANOVA analysis of variance was used to evaluate significant differences. Data were graphically presented using GraphPad Prism 6 (GraphPad, San Diego CA). The *P value* of less than 0.05 was considered statistically significant.

## Abbreviations

CSD, Chronic sleep disturbance; TMJ-OA, temporomandibular joint osteoarthritis; TMJ, temporomandibular joint; TMDs, temporomandibular disorders; VEGF, vascular endothelial growth factor; Dll4, Delta-like 4; p-ERK1/2,Phospho-ERK1/2; MMPM, modified multiple platform method; MCC, mandibular condylar cartilage; CON, the control; R, recovery; CORT, serum corticosterone; ACTH, adrenocorticotropic hormone; HE, haematoxylin and eosin; MVD, microvessel density.
